# Posttraumatic stress disorder factor structure in hurricane‐affected Puerto Ricans: A PTSD Checklist for *DSM‐5* comparison with non‐Latiné White individuals

**DOI:** 10.1002/jts.70016

**Published:** 2025-09-16

**Authors:** Johanna E. Hidalgo, Keith B. Burt, Tatiana M. Davidson, Kenneth J. Ruggiero, Arthur R. Andrews, Ateka A. Contractor, Kelly Peck, Ellen W. McGinnis, Jennifer Ha, Natalie C. Noble, Julia N. Kim, Vanessa Ramirez, Matthew Price

**Affiliations:** ^1^ Department of Psychological Science University of Vermont Burlington Vermont USA; ^2^ Department of Nursing Medical University of South Carolina Charleston South Carolina USA; ^3^ SmartState Technology Application Center for Healthful Lifestyles Medical University of South Carolina Charleston South Carolina USA; ^4^ Department of Psychology and Institute for Ethnic Studies University of Nebraska–Lincoln Lincoln Nebraska USA; ^5^ College of Liberal Arts and Social Sciences University of North Texas Denton Texas USA; ^6^ Social Sciences and Health Policy Wake Forest University Winston‐Salem North Carolina USA

## Abstract

Due to Puerto Rico's location, there is heightened vulnerability to the consequences of natural disasters, contributing to an elevated risk of posttraumatic stress disorder (PTSD). Given PTSD's heterogeneous nature, this study examined whether PTSD factor structure, based on *DSM‐5* criteria and measured using the PTSD Checklist for *DSM‐5* (PCL‐5), was equivalent across hurricane‐exposed Puerto Ricans (*n* = 596) and non‐Latiné White (NLW) individuals (*n* = 459). Confirmatory factor analysis (CFA) indicated the seven‐factor hybrid model of PTSD was the best‐fitting structure, χ^2^(*N* = 897, 298) = 685.59, CFI = .967, TLI = .958, RMSEA = .054, SRMR = .038. Latent factor correlations (range: .61–.93) supported the distinctiveness of PTSD symptom dimensions. PTSD prevalence estimates varied significantly (*DSM‐5*: 47.8%, hybrid: 28.2%). Multigroup CFA results supported partial scalar invariance, with PCL‐5 Item 8 (memory impairment) requiring varying intercepts, χ^2^(*N* = 897, 330) = 806.97, *p* < .001, CFI = .960, TLI = .954, RMSEA = .057, 90% CI [.052, .062], SRMR = .047, BIC = 49,586.9. NHWs reported higher avoidance (Δ*M* = 0.186), *p* = .011; negative affect (Δ*M* = 0.160), *p* = .028; anhedonia (Δ*M* = 0.217), *p* = .002; and dysphoric arousal symptoms (Δ*M* = 0.187), *p* = .015, relative to Puerto Ricans. Strong associations between PTSD factors and depression and psychological distress, βs = .57–.82, supported convergent validity. Findings highlight the relevance of the hybrid model for conceptualizing PTSD symptoms among hurricane‐exposed populations, with important implications for culturally informed assessment and treatment in Puerto Rican communities.

Puerto Rico's location makes residents vulnerable to natural disasters, with 56% of its population residing in coastal areas (Padua Soto, 2024). Hurricane María in 2017 caused an estimated 2,975 deaths and displaced over 200,000 Puerto Ricans (García‐López, 2018; Milken Institute School of Public Health, 2018). Federal disaster relief was insufficient, with 2017's Hurricane Harvey in Texas receiving $141,800,000 (USD) in Federal Emergency Management Agency (FEMA) aid within 9 days compared to only $6,200,000 for Puerto Rico (García‐López, 2018). The combination of massive casualties, displacement, insufficient federal response, and infrastructure collapse created prolonged trauma exposure and secondary stressors, including increased drug use, homicides, and domestic violence (Abrams, 2019). These factors likely contributed to exceptionally high posttraumatic stress disorder (PTSD) rates among survivors, ranging from 41.9% to 65.7% among displaced residents (Scaramutti et al., 2019), with 24.3% of survivors in one survey reporting severe symptom levels (Ferré et al., 2019). Although the prevalence of PTSD in Puerto Ricans after Hurricane María is well documented, the latent factor structure of PTSD symptoms, as outlined in the *Diagnostic and Statistical Manual of Mental Disorders* (5th ed.; *DSM‐*5; American Psychiatric Association [APA], 2013) and assessed using the PTSD Checklist for *DSM‐5* (PCL‐5; Bovin et al., 2016; Weathers et al., 2013), has not been rigorously examined. It remains unclear whether the factor structure of PTSD observed predominantly in Non‐Latiné White (NLW) samples aligns with Puerto Rican individuals. Given the heterogeneous nature of PTSD (Galatzer‐Levy & Bryant, 2013) and Puerto Rico's disaster vulnerability, it is crucial to determine whether the PCL‐5 accurately captures Puerto Rican symptom presentations to inform culturally appropriate assessments and interventions.

Recent research suggests there are multiple possible factor structures for PTSD. The most widely used is the *DSM‐5*'s four‐factor PTSD model (APA, 2013). Two alternative models have also received empirical support: (a) the *anhedonia model* (Liu et al., 2014) and (b) the *hybrid model* (Armour et al., 2015). Both models distinguish between negative affect (e.g., negative beliefs, blame) and anhedonia (e.g., loss of interest, detachment). The anhedonia and hybrid models share six core factors: intrusion, avoidance, negative affect, anhedonia, dysphoric arousal, and anxious arousal. However, the hybrid model incorporates a seventh factor: externalizing behaviors. Work with majority‐NLW samples has supported the superior fit of the hybrid model.

The factor structure of PTSD may differ for Puerto Ricans compared with NLW populations. PTSD prevalence among Puerto Ricans ranges from 6.7% to 43.6% (Torres‐Valentin et al., 2021), differing from the 8.7% in the general U.S. population, potentially reflecting differences in symptom presentation. Ortega and Rosenheck (2000) documented that mainland Puerto Ricans exhibited higher rates of avoidance symptoms. Culturally specific expressions of distress among Puerto Ricans may also impact the relations among symptoms. Adversity‐related constructs reported among Puerto Rican samples—such as *susto*, *nervios*, and *ataques de nervios*—manifest as somatic complaints, dissociative episodes, or intense emotional expressions (Guarnaccia et al., 1993). These expressions may contribute to distinct PTSD presentations, symptom severity, and factor structure (Baer, 2003). For example, one study showed that Puerto Ricans express distress with greater emphasis on somatic complaints rather than cognitive symptoms (Ruef et al., 2000), which could reflect different associations between *DSM‐5* Cluster D (i.e., negative cognitions in cognitions and mood) and Cluster E (i.e., alterations in arousal and reactivity) symptoms. Marshall and Orlando (2002) also found elevated peritraumatic dissociation in individuals with stronger adherence to Latiné cultural norms, potentially influencing Cluster D symptoms. Thus, the elevated prevalence of PTSD in Puerto Rican individuals may result from differences in symptom severity and differences in the underlying factor structure.

Cross‐cultural research suggests that the PTSD factor structure varies across international samples. In Mexico, the six‐factor anhedonia model fit well among Spanish‐speaking earthquake survivors (Durón‐Figueroa et al., 2019). In Spain, both the anhedonia and hybrid models were found to fit, though the externalizing behaviors factor from the hybrid model failed to demonstrate cohesion as a distinct factor (Soberón et al., 2016). In contrast, U.S. community samples have supported the seven‐factor hybrid model (Armour et al., 2015; Seligowski & Orcutt, 2016). Among disaster‐exposed populations, studies with Filipino relocatees and typhoon Haiyan survivors supported the seven‐factor hybrid model over alternatives (Mordeno & Hall, 2017; Mordeno et al., 2016). In Korean adults exposed to earthquakes and floods, both the anhedonia and hybrid models showed comparable fit (Seo & Cho, 2023). Taken together, these findings indicate that PTSD symptom organization may differ across cultural groups such that the specific factor structure may vary based on cultural expressions of distress.

As a predominantly bilingual population frequently affected by natural disasters (Carroll & Ramos, 2024), island‐based Puerto Ricans may express and organize PTSD symptoms in ways not captured by models validated in mainland Latiné or general U.S. populations. This study aimed to evaluate the fit of three PTSD models (*DSM‐5*, anhedonia, and hybrid) and access model invariance across disaster‐affected Puerto Ricans and NLW populations. We hypothesized that the hybrid model would demonstrate superior fit compared to the *DSM‐5* and anhedonia models. We expected the factor structure and factor loadings would be similar across groups (metric invariance) but anticipated differences in item thresholds between Puerto Rican and NLW individuals (lack of scalar invariance). Convergent validity was evaluated by testing whether PTSD factors showed similar patterns of association with depression (Patient‐Reported Outcomes Measurement Information System Depression Short‐Form [PROMIS‐D‐SF; Pilkonis et al., 2011]) and psychological distress (Kessler Screening Scale for Psychological Distress [K6; Kessler et al., 2002]) across groups. Through this systematic approach, we evaluated both the measurement equivalence of PTSD symptoms and their associations with related constructs across Puerto Rican and NLW populations. To evaluate model distinctiveness and clinical implications, we examined (a) interfactor correlations within each model to assess the distinctiveness of symptom dimensions; (b) prevalence estimates of probable PTSD using model‐specific thresholds; and (c) latent mean differences across groups to detect differences in the estimated factor means, reflecting potential variation in underlying symptom dimensions.

## METHOD

### Participants and procedure

Participants were recruited through a larger web‐based intervention study. Social media advertisements targeted individuals in zip codes affected by Hurricanes Harvey (Texas), Irma (Florida), Florence (North Carolina), Maria (Puerto Rico), and Michael (Florida and Georgia) during 2017–2018. Eligible participants were English‐speaking adults aged 18–78 years with internet access in the affected regions. Due to limitations of this secondary data analysis, participant heritage information was unavailable. This study included data from 1,055 participants: Puerto Rican hurricane survivors (*n* = 596) and NLW individuals (*n* = 459). Latiné participants who did not reside in Puerto Rico and individuals from other racial backgrounds (*n* = 302, 22.3%) were excluded. Table 1 presents demographic information. The Medical University of South Carolina Institutional Review Board approved this study (NCT03403738).

**TABLE 1:** Characteristics of Puerto Ricans and Non‐Latiné White participants

	Puerto Ricans				Non‐Latiné White				Total sample			
	(*n* = 596)				(*n* = 459)				(*n* = 1,055)			
Variable	*n*	%	*M*	*SD*	*n*	%	*M*	*SD*	*n*	%	*M*	*SD*
Age (years)			43.78	13.25			44.13	12.36			43.93	12.86
Gender												
Woman	461	77.3			380	82.8			841	79.7		
Man	135	22.6			74	16.1			209	19.8		
Identify with other gender	0	0.0			5	1.1			5	0.5		
Income (USD)												
< $20,000	290	48.7			194	42.2			484	45.9		
≥ $20,000^b^	262	44.0			249	54.2			511	48.4		
Prefer not to answer	44	7.4			16	3.5			60	5.7		
Educational attainment												
≤ High school diploma	57	9.6			133	29.0			190	18.0		
> High school diploma^b^	531	89.0			280	61.0			811	76.9		
Prefer not to answer	8	1.0			2	0.4			10	0.9		
Hurricane exposure												
Harvey (Texas)					64	13.9			64	6.1		
Maria (Puerto Rico)	596	100.0			51	11.1			647	61.3		
Florence (North Carolina)					43	9.4			43	4.1		
Irma (Florida)					31	6.8			31	2.9		
Michael (Florida, Georgia)					270	58.8			270	25.6		
Partner status												
With partner	287	48.1			246	53.6			533	50.5		
Single	287	48.1			207	45.1			494	46.8		
Prefer not to answer	22	3.7			6	1.3			28	2.7		
PCL‐5^b^			36.17	18.72			38.95	18.40			37.53	18.61
PROMIS‐D‐SF^b^			22.18	8.86			25.33	8.57			23.55	8.87
K6^c^			6.13	5.10			7.53	4.89			6.73	5.05

*Note*. PCL‐5 = PTSD Checklist for *DSM‐5*; PROMIS‐D‐SF = Patient‐Reported Outcomes Measurement Information System Depression Short‐Form; K6 = Kessler Screening Scale for Psychological Distress.

^a^ Range: 18–78 years.

^b^ Between‐group comparison significant at *p* < .01.

^c^ Between‐group comparison significant at *p* < .001.

Most participants were women (79.7%), with a mean age of 43.93 years (*SD =* 12.86). Age did not differ significantly between NLW and Puerto Rican participants, *t*(1,015) = 0.44, *p* = .659. Although most participants had at least a high school diploma, Puerto Rican participants were significantly more likely than NLW participants to have an educational attainment beyond high school, χ^2^(1, *N* = 995) = 63.82, *p* < .001. In contrast, NLW participants had significantly higher household incomes than Puerto Rican participants, χ^2^(1, *N* = 995) = 7.18, *p* = .007 (see Table 1).

### Measures

#### PTSD symptoms

The PCL‐5 (Weathers et al., 2013) is a 20‐item, self‐report measure that was used to assess past‐month *DSM‐5* PTSD symptoms. Participants rated symptoms in relation to the recent hurricane they experienced, scoring responses on a scale of 0 (*not at all*) to 4 (*extremely*). Total scores range from 0 to 80, with a score of 31 or higher indicating probable PTSD. The PCL‐5 showed excellent internal consistency in the original validation study (Cronbach's α = .96; Bovin et al., 2016). In the current study, the scale showed excellent internal consistency for Puerto Rican participants, Cronbach's α = .95, and NLW participants, Cronbach's α = .95.

#### Depressive symptoms

The PROMIS‐D‐SF (Pilkonis et al., 2011) is an eight‐item, self‐report measure that was used to assess depressive symptoms in the prior 7 days. Participants were asked to rate items on a 5‐point Likert scale ranging from 1 (*never*) to 5 (*always*), with total scores ranging from 8 to 40; scores of 15 or higher indicate clinically significant depressive symptoms. The PROMIS‐D‐SF showed excellent internal consistency in the original validation study (Cronbach's α = .95). In the current study, the scale showed excellent internal consistency for Puerto Rican participants, Cronbach's α = .96, and NLW participants, Cronbach's α = .96.

#### Psychological distress

The K6 (Kessler et al., 2002) is a six‐item self‐report measure that was used to assess the frequency and severity of past‐month psychological distress symptoms. Items assess six symptoms: feelings of nervousness, hopelessness, restlessness, depression, worthlessness, and effortfulness in daily life. Responses are rated on a scale of 0 (*none of the time*) to 4 (*all the time*). Total scores range from 0 to 24, with scores of 13 or higher indicating probable serious mental illness. The K6 showed excellent internal consistency in the original validation study (Cronbach's α = .86). In the current study, the scale showed excellent internal consistency for Puerto Rican participants, Cronbach's α = .91, and good consistency for NLW participants, Cronbach's α = .89.

### Data analysis

Confirmatory factor analyses (CFAs) were conducted using the *lavaan* package in R (Version 4.2.2) for structural equation modeling. Maximum likelihood estimation was used due to its efficiency under multivariate normality (Enders, 2010), and missing data were handled using full information maximum likelihood (Enders & Bandalos, 2001). Covariance coverage was 1.00 across all observed variable pairs in each proposed model and .85 in models including PROMIS‐D‐SF and K6 scores, indicating no pairwise missingness and supporting reliable model estimation. Model fit was assessed based on recommendations from Hu and Bentler (1999) such that adequate fit required a standardized root mean square residual (SRMR) value of .08 or lower and at least one of the following: a comparative fit index (CFI) value of .95 or greater, Tucker–Lewis index (TLI) value of .95 or greater, or root mean square error of approximation (RMSEA) value of .06 or less.

To evaluate the PTSD factor structure, competing models were tested within each racial/ethnic group, then in a multigroup framework. When supported across groups, configural invariance was tested to assess structural equivalence (Little & Slegers, 2005). The *DSM‐5* structure was included in invariance testing comparisons, as it is the current diagnostic standard.

Following model selection, measurement invariance was examined using a commonly applied framework (e.g., Contractor et al., 2019). This approach involves progressively stringent criteria: configural invariance (similar factor structure), metric invariance (similar factor loadings), scalar invariance (similar item intercepts), and strict invariance (similar residual variances; Milfont & Fisher, 2010). Metric and scalar invariance were tested through comparisons of constrained and unconstrained models. To assess noninvariance, changes in model fit indices were evaluated based on based on criteria outlined by Chen (2007). Metric noninvariance was indicated by a CFI change of .010 or greater, RMSEA change of .015 or greater, or SRMR change of .030 or greater, whereas intercept or residual noninvariance was identified using a CFI change of .010 or greater, RMSEA change of .015 or greater, or SRMR change of .010 or greater. Metric invariance was tested by constraining factor loadings and comparing the model to the unconstrained version, whereas scalar invariance was examined by further constraining intercepts. Partial scalar invariance was evaluated when scalar invariance was not achieved by freeing the intercept of the item with the largest group difference (Vandenberg & Lance, 2000). Once partial scalar invariance was established, latent mean differences were examined by setting the NLW group's mean to 0 and estimating latent means for the Puerto Rican group. This approach ensured that observed differences reflected true latent differences rather than measurement bias (Milfont & Fischer, 2010).

To ensure robust model comparisons, chi‐square difference tests and Bayesian information criterion (BIC) were used to compare model fit between constrained and unconstrained models. Invariance was supported when the chi‐square difference test was nonsignificant (i.e., a *p* value of .05 or greater; Little, 2013) and when the change in CFI remained below 0.01 (Cheung & Rensvold, 2002; Contractor et al., 2018).

PTSD prevalence was estimated using a model‐based diagnostic approach consistent with prior research (Murphy et al., 2018; Shevlin et al., 2017). PCL‐5 responses were dichotomized (i.e., endorsement was considered to be a rating of 2 or higher) and grouped by model factor structure. Probable PTSD required the endorsement of one or more symptoms in clusters with two to five items and two or more symptoms in clusters with six or more items (i.e., six of 20 symptoms for *DSM‐5*, seven of 20 for the hybrid model).

## RESULTS

### Psychopathology comparisons

NLW participants reported significantly higher levels of PTSD symptom severity than Puerto Rican participants, *t*(894.44) = 2.24, *p* = .025. NLW participants also endorsed significantly higher levels of depressive symptoms, *t*(1,000.5) = 5.83, *p* < .001, and psychological distress, *t*(1,003.7) = 4.52, *p* < .001, than their Puerto Rican counterparts (Table 1).

### Single‐group CFA between ethnic groups

The hybrid model performed best for both Puerto Rican, χ^2^(149, *N* = 458) = 347.82, CFI = .968, TLI = .959, RMSEA = .054, 90% confidence interval (CI) [.047, .061], SRMR = .044, and NLW participants, χ^2^(149, *N* = 439) = 337.77, CFI = .966, TLI = .957, RMSEA = .054, 90% CI [.046, .061], SRMR = .036 (Table 2).

**TABLE 2:** Fit indices of the single‐group confirmatory factor analyses of posttraumatic stress disorder in the Puerto Rican and Non‐Latiné White subsamples

Model	χ^2^	*df*	CFI	TLI	AIC	BIC	RMSEA	90% CI	SRMR
Puerto Rican									
*DSM‐5*	614.26^***^	190	.928	.916	25,011.0	25,200.1	.077	[.071, .084]	.055
Anhedonia	403.06^***^	155	.960	.951	24,817.8	25,044.8	.059	[.052, .066]	.047
Hybrid	347.82^***^	149	.968	.959	24,774.6	25,026.7	.054	[.047, .061]	.044
Non‐Latiné White									
*DSM‐5*	646.41^***^	164	.914	.901	24,329.6	24,517.5	.082	[.075, .089]	.052
Anhedonia	399.78^***^	155	.957	.947	24,100.9	24,325.6	.060	[.053, .067]	.060
Hybrid	337.77^***^	149	.966	.957	24,050.9	24,300.1	.054	[.046, .061]	.036

*Note*: *N* = 1,055, Puerto Ricans: *n* = 672, Non‐Latiné Whites: *n* = 459. *DSM‐5* = *Diagnostic and Statistical Manual of Mental Disorders* (5th ed.); *df* = degrees of freedom; AIC = Akaike information criterion; CFI = comparative fit index; TLI = Tucker–Lewis index; RMSEA = root mean square error of approximation; CI = confidence interval; SRMR = standardized root mean square residual; BIC = Bayesian information criterion.

^***^ *p* < .001.

### Hybrid model fit and DSM‐5 invariance across ethnic groups

Multigroup CFA (MGCFA) revealed that the hybrid model demonstrated superior fit, χ^2^(298, *N* = 897) = 685.59, CFI = .967, TLI = .958, RMSEA = .054, 90% CI [.049, .059], SRMR = .038, compared to both the *DSM‐5* and Anhedonia models (Table 3). Invariance was evaluated first with the *DSM‐5* model. Model fit statistics supported configural, metric, scalar, and strict invariance across groups (see Table 4). Metric invariance was supported with acceptable fit, χ^2^(348, *N* = 897) = 1,292.72, RMSEA = .078, 90% CI [.073, .082], CFI = .920, TLI = .913. Scalar invariance held when factor loadings and intercepts were constrained, χ^2^(364, *N* = 897) = 1,411.07, RMSEA = .080, 90% CI [.076, .085], CFI = .912, TLI = .908. Strict invariance was supported when residual variances were constrained, χ^2^(384, *N* = 897) = 1442.45, RMSEA = .078, 90% CI [.074, .083], CFI = .911, TLI = .912. Overall, strict invariance had the highest level of measurement equivalence and was supported for the *DSM‐5* model.

**TABLE 3:** Posttraumatic stress disorder unconstrained multigroup confirmatory factor analysis indices of fit

Model	χ^2^	*df*	CFI	TLI	AIC	BIC	RMSEA	90% CI	SRMR
*DSM‐5*	1260.67^***^	328	.921	.909	49,420.6	50,054.1	.080	[.075, .084]	.051
Anhedonia	802.84^***^	310	.959	.949	48,998.8	49,718.6	.060	[.054, .065]	.042
Hybrid	685.59^***^	298	.967	.958	48,905.5	49,682.9	.054	[.049, .059]	.038

*Note*: *N* = 1,055. *DSM‐5* = *Diagnostic and Statistical Manual of Mental Disorders* (5th ed.); *df* = degrees of freedom; AIC = Akaike information criterion; CFI = comparative fit index; TLI = Tucker–Lewis index; RMSEA = root mean square error of approximation; CI = confidence interval; SRMR = standardized root mean square residual; BIC = Bayesian information criterion.

^***^ *p* < .001.

**TABLE 4:** Posttraumatic stress disorder model measurement invariance indices of fit

Model and measurement invariance	χ^2^	*df*	CFI	TLI	AIC	BIC	RMSEA	90% CI	SRMR
*DSM‐5* model									
Metric	1,292.72^***^	348	.920	.913	49,412.6	49,950.1	.078	[.073, .082]	.060
Scalar	1,411.07^***^	364	.912	.908	49,499.0	49,959.7	.080	[.076, .085]	.063
Strict	1,442.45^***^	384	.911	.912	49,490.4	49,855.1	.078	[.074, .083]	.062
Hybrid model									
Metric	709.37^***^	318	.967	.961	48,889.3	49,570.8	.052	[.047, .058]	.045
Scalar	852.72^***^	311	.958	.952	48,979.6	49,598.7	.058	[.053, .063]	.048
Partial scalar	806.973^***^	330	.960	.954	48,962.9	49,586.9	.057	[.052, .062]	.047
Strict	844.66^***^	351	.958	.955	48,958.6	49,481.8	.056	[.051, .061]	.056

*Note*: *N* = 1,055. *DSM‐5* = *Diagnostic and Statistical Manual of Mental Disorders* (5th ed.); *df* = degrees of freedom; AIC = Akaike information criterion; CFI = comparative fit index; TLI = Tucker–Lewis index; RMSEA = root mean square error of approximation; CI = confidence interval; SRMR = standardized root mean square residual; BIC = Bayesian information criterion.

^***^ *p* < .001.

### Measurement invariance for the hybrid model

Next, measurement invariance was evaluated with the hybrid model between both ethnic groups. The criteria were met for configural, metric, and partial scalar invariance (Table 4). The model demonstrated metric invariance, with a nonsignificant chi‐square difference test, Δχ^2^(20) = ‐39.18, *p* = 1.00, ΔCFI = .000. When testing scalar invariance, there was a significant worsening in model fit, Δχ^2^(13) = 116.35, *p* < .001, ΔCFI = .009. An examination of modification indices revealed that PCL‐5 Item 8 (memory impairment) had the largest intercept difference between groups. A partial scalar invariance model was estimated with Item 8's intercept freed across groups. This model showed improved fit. Although the chi‐square difference test comparing the partial scalar to the metric model was significant, Δχ^2^(12) = 97.60, *p* < .001, the CFI difference remained below .01, supporting partial scalar invariance.

### Latent mean differences for the hybrid model

After establishing partial scalar invariance, we examined latent mean differences using the Puerto Rican group as the reference (means constrained to 0). NLW participants demonstrated significantly higher levels of severity for avoidance (Δ*M* = 0.186), *p* = .011; negative affect (Δ*M* = 0.160), *p* = .028; anhedonia (Δ*M* = 0.217), *p* = .002; and dysphoric arousal symptoms (Δ*M* = 0.187), *p* = .015, compared to Puerto Rican participants. No statistically significant differences were observed for intrusion (Δ*M* = 0.137), *p* = .053; externalizing behaviors (Δ*M* = 0.133), *p* = .094); or anxious arousal symptoms (Δ*M* = 0.077), *p* = .294.

### Psychological distress and depression: Hybrid model covariates

A MGCFA was conducted to examine differences in the covariance between PROMIS‐D‐SF scores and the latent factors for the hybrid model of PTSD (Table 5). The model demonstrated good fit at the configural, CFI = .968, RMSEA = .053, 90% CI [.048, .058], and metric levels, CFI = .968, RMSEA = .052, 90% CI [.047, .057], with minimal change in fit indices, ΔCFI = .000, ΔRMSEA = ‐.001. Whereas scalar invariance showed some worsening in fit, CFI = .958, RMSEA = .058, 90% CI [.053, .063], partial scalar invariance demonstrated the best fit, CFI = .960, RMSEA = .057, 90% CI [.052, .061]. When equality constraints were placed on the covariances, the model showed minimal change in fit, ΔCFI = ‐.001, ΔRMSEA = ‐.001, ΔSRMR = .005.

**TABLE 5:** Depression, psychological distress, and hybrid model measurement invariance indices of fit

Model and measurement invariance	χ^2^	*df*	CFI	TLI	AIC	BIC	RMSEA	90% CI	SRMR
PROMIS‐D‐SF									
Configural	735.17^***^	324	.968	.959	54,404.7	55,268.5	.053	[.048, .058]	.037
Metric	758.92^***^	344	.968	.961	54,388.5	55,156.3	.052	[.047, .057]	.045
Scalar	902.05^***^	358	.958	.950	54,503.6	55,204.3	.058	[.053, .063]	.056
Partial scalar	871.49^***^	357	.960	.953	53,383.9	54,089.4	.057	[.052, .061]	.057
Strict	909.55^***^	379	.959	.955	53,378.0	53,977.9	.056	[.051, .061]	.054
K6									
Configural	730.69^***^	324	.969	.959	53,309.1	54,172.8	.053	[.048, .058]	.037
Metric	754.28^***^	344	.968	.961	53,292.7	54,060.6	.052	[.047, .057]	.044
Scalar	890.08^***^	358	.956	.952	53,400.5	54,101.2	.058	[.053, .062]	.054
Partial scalar	883.52^***^	357	.959	.952	54,487.1	55,192.5	.057	[.053, .062]	.055
Strict	920.69^***^	379	.958	.953	54,480.2	55,080.1	.056	[.052, .061]	.055

*Note*: *N* = 1,055; PROMIS‐D‐SF = Patient‐Reported Outcomes Measurement Information System Depression Short‐Form; K6 = Kessler Screening Scale for Psychological Distress; *df* = degrees of freedom; AIC = Akaike information criterion; CFI = comparative fit index; TLI = Tucker–Lewis index; RMSEA = root mean square error of approximation; CI = confidence interval; SRMR = standardized root mean square residual; BIC = Bayesian information criterion.

^***^ *p* < .001.

An additional analysis with K6 scores showed similar patterns, with good fit at the configural, CFI = .969, RMSEA = .053, 90% CI [.048, .058], SRMR = .037, and metric levels, CFI = .968, RMSEA = .052, 90% CI [.047, .057], SRMR = .044. After scalar invariance showed a degradation in fit, CFI = .956, RMSEA = .058, partial scalar invariance was tested by freeing PCL‐5 Item 8, resulting in an improved fit, CFI = .959, RMSEA = .057, 90% CI [.053, .062], SRMR = .055, and adding equality constraints on the covariances resulted in minimal changes to model fit, ΔCFI = ‐.001, ΔRMSEA = ‐.001, ΔSRMR = .005. Standardized covariances between the hybrid model's PTSD factors and PROMIS‐D‐SF and K6 scores ranged from .57 to .82 across both groups. These represented large effect sizes according to Cohen's (1988) conventions, indicating strong associations between PTSD symptom domains and symptoms of both depression and distress. The strongest associations were observed for anhedonia, negative affect, and dysphoric arousal, βs = .79–.82.

### Prevalence estimates

PTSD prevalence estimates differed significantly across the two models. In the full sample, the *DSM‐5* model yielded a significantly higher estimated prevalence (47.8%) than the hybrid model (28.2%), *z* = 9.29, *p* < .001. When stratified by ethnicity, a similar pattern emerged among Puerto Rican participants such that the *DSM‐5* model produced a significantly higher prevalence than the hybrid model (40.4% vs. 27.7%); *z* = 4.64, *p* < .001. Among NLW participants, the *DSM‐5* model again yielded a significantly higher estimate (57.3%), relative to the hybrid model (28.2%), *z* = 8.87, *p* < .001.

### Correlations between models

Correlations among latent factors were examined to assess overlap and distinctiveness within the *DSM‐5* and hybrid model. See Table 6 for all correlation values.

**TABLE 6:** Factor correlations for the hybrid and *DSM‐5* models

Model and factor	INT	AVD	NACM	AAR
*DSM‐5*				
INT	–	.93^***^	.73^***^	.76^***^
AVD		–	.78^***^	.78^***^
NACM			–	.89^***^
AAR				–

	INT	AVD	NAF	ANH	EXT	AXA	DAR
Hybrid							
INT	–	.93^***^	.76^***^	.66^***^	.61^***^	.73^***^	.70^***^
AVD		–	.79^***^	.73^***^	.65^***^	.73^***^	.72^***^
NAF			–	.86^***^	.83^***^	.69^***^	.76^***^
ANH				–	.85^***^	.67^***^	.85^***^
EXT					–	.74^***^	.74^***^
AXA						–	.80^***^
DAR							–

*Note*: *DSM‐5* = *Diagnostic and Statistical Manual of Mental Disorders* (5th ed.); AAR = alterations in arousal and reactivity; ANH = anhedonia; AVD = avoidance; AXA = anxious arousal; DAR = dysphoric arousal; EXT = externalizing behaviors; INT = intrusions; NAF = negative affect; NACM = negative alterations in cognition and mood.

^***^ *p* < .001.

## DISCUSSION

This study identified the best‐fitting PTSD factor structure for Puerto Rican and NLW hurricane survivors. Results of the MGCFA indicated that the seven‐factor hybrid model had significantly better overall fit. The *DSM‐5* model, despite poorer fit indices, achieved strict measurement invariance, suggesting comparable measurement properties across both Puerto Rican and NLW hurricane survivors. This finding contrasts with previous studies in Spanish‐speaking Latiné populations supporting the anhedonia model (Durón‐Figueroa et al., 2019; Soberón et al., 2016). Measurement invariance testing for the hybrid model achieved partial scalar invariance. One item required being freed across groups (i.e., Item 8, memory impairment). This finding is consistent with previous research documenting psychometric issues with this item, including weaker associations with other PTSD symptoms and inconsistent factor loadings (Berntsen & Rubin, 2014). These results highlight the benefit of population‐specific validation studies rather than assuming generalizability across diverse Latiné subgroups.

The hybrid model was retained for its superior fit. Latent mean comparisons revealed that NLW participants reported significantly higher PTSD symptom severity than Puerto Rican participants for avoidance, negative affect, anhedonia, and dysphoric arousal symptoms. No significant differences emerged for intrusion, externalizing behaviors, or anxious arousal. These findings suggest that the hybrid model allows for more precision in detecting group‐level differences in specific symptom domains that may have clinical relevance. In contrast, the *DSM‐5* model showed significant differences only for avoidance (Cluster B) and negative alterations in cognition and mood (Cluster D). The hybrid model showed higher interfactor distinctiveness, with *r* values for 81% of correlations below .85 (Brown, 2015), suggesting relatively distinct latent factors. The enhanced differentiation of the hybrid model suggests this model may have theoretical and psychometric value in capturing nuanced PTSD symptom dimensions.

The different factor structure and associated diagnostic framework may reflect a difference in the utility of each framework. PTSD is a highly heterogeneous diagnosis (Galatzer‐Levy & Bryant, 2013), which can prove challenging when identifying treatment targets. From a clinical perspective, the hybrid model may provide greater specificity for individual presentations than the *DSM‐5* model for individual presentations, thereby supporting more targeted treatment. Following Hurricane Maria, Puerto Rico saw increases in homicides, domestic abuse, and substance use (Abrams, 2019). Identifying elevated externalizing behaviors, which are more prominent after these types of trauma, using the hybrid model may help clinicians tailor support strategies. Conversely, the *DSM‐5* model has increased sensitivity given that it requires the presence of fewer symptoms that are less distributed across the 20 possible PTSD symptoms. The *DSM‐5* model may prove more useful for estimating postdisaster PTSD incidence to determine community resource needs. Future work is required to evaluate the utility of each of these frameworks in contexts where access to care (e.g., postdisaster services, community screening) is needed or where the priority is accurate case conceptualization (e.g., research or specialized clinical settings).

Although Puerto Ricans are often identified as being at higher risk for adverse mental health outcomes due to structural inequities and disaster exposure (García‐López, 2018), our findings showed higher PTSD, depressive, and psychological distress symptom scores among NLW participants. This contrasts with prior research indicating higher levels of symptom severity among Latiné populations (e.g., Perilla et al., 2002; Torres‐Valentin et al., 2021). Several factors may explain this unexpected pattern. First, NLW participants in our sample had a lower average level of educational attainment, a known risk factor for depression and psychological distress (Kondirolli & Sunder, 2022; Muñoz & Santos‐Lozada, 2021). Second, a prior study found that the association between disaster‐related service loss and PTSD symptoms was stronger among NLW participants than Latiné participants (Davidson et al., 2013), suggesting that disaster‐related stressors may differentially affect groups depending on contextual vulnerabilities rather than ethnicity alone. Third, Puerto Rican participants may have benefited from culturally embedded coping resources, such as family‐based social support, which is more prominent in Latiné communities and linked to lower distress (Sabogal et al., 1987). Additionally, culturally shaped symptom expression may have contributed to lower observed scores among Puerto Rican participants, as bilingual individuals may experience higher levels of emotional depth when discussing distressing experiences in Spanish (Altarriba, 2003; Javier et al., 1993), potentially affecting symptom reporting in English‐administered assessments. Patterns of distress‐underreporting among Latiné populations, influenced by social desirability concerns, may also contribute to lower observed PTSD symptom severity despite meaningful underlying distress (Pole et al., 2005). Thus, although Puerto Ricans remain at systemic risk, the group‐level differences in symptom severity observed here likely reflect the complex intersection of disaster exposure, social determinants of health, resilience factors, and culturally specific manifestations of distress.

To assess convergent validity, we examined associations between each hybrid model PTSD factor and two theoretically related measures of internalizing symptoms: Depressive symptoms (PROMIS‐D‐SF) and psychological distress (K6). Standardized covariances with these measures ranged from .57 to .82 across Puerto Rican and NLW participants, indicating large effect sizes (Cohen, 1988). The strongest associations were observed for anhedonia, negative affect, and dysphoric arousal, βs = .79–.82, consistent with theoretical expectations that these domains are closely tied to depressive and distress‐related symptoms (Armour et al., 2015; Claycomb Erwin et al., 2017) and may be especially relevant for clinical interpretation. These results support the hybrid model's convergent validity and align with Rasmussen et al.’s (2019) recommendation to examine how PTSD factors relate to external constructs to enhance their clinical and construct validity. Notably, MGCFA results showed that the covariances between PTSD factors and external measures were comparable across Puerto Rican and NLW participants, providing further support for the stability of these associations.

Recent work has critiqued the complexity of PTSD factor structure models. Specifically, Rasmussen et al. (2019) and Shevlin et al. (2017) highlight the lack of clinical utility of such models and the inclusion of factors that contain fewer than the recommended (i.e., two) indicators. Such factors can be statistically unstable and conceptually ambiguous. In support of these more complex factor structures, the present study's finding of invariance among the interfactor correlations and latent means across two ethnically distinct groups contributes to the evidence in support of the hybrid model. However, these models provide substantially different diagnosis rates (*DSM‐5*: 47.8%, hybrid: 28.2%), which is consistent with other studies (Shelvin et al., 2017). The difference in diagnostic rates is attributed to the diagnostic algorithms, which require a higher threshold for meeting diagnostic criteria by the hybrid model diagnostic algorithm. The *DSM‐5* model requires a minimum of six symptoms to meet the criteria for PTSD, whereas the hybrid model requires a minimum of seven symptoms. The hybrid model requires a broader spread of symptoms than the *DSM‐5* model, requiring at least one symptom per cluster. It is unclear if this difference is due solely to changes in the sensitivity and specificity of this diagnostic algorithm or if it reflects a true change in the prevalence of PTSD. Future work should explore alternative diagnostic algorithms for PTSD using the hybrid model that require subsets of the additional five factors created from *DSM‐5* Clusters D and E, similar to the algorithmic method of depression diagnoses. This work may reveal that an alternative diagnostic algorithm allows for balancing the utility of a sensitive and specific diagnostic rate that offers improved clarity regarding a given individual's symptom presentation.

The precision of the hybrid model becomes particularly relevant when viewed through the lens of cultural variations in trauma response. Among Puerto Ricans and broader Latiné populations, avoidance‐oriented coping has been linked to fatalistic beliefs, defined as a spiritual or stoic acceptance of adversity, which can hinder engagement in care and complicate recovery (Abraído‐Lanza et al., 2007; Perilla et al., 2002). Trauma may be interpreted through religious or moral frameworks, such as divine punishment, associated with elevated PTSD risk and inadequate preparedness for future disasters (O'Connell et al., 2017; Rahmani et al., 2022). The hybrid model's distinction between avoidance, anhedonia, and dysregulated affect may help clinicians better identify culturally grounded patterns of distress. Interventions that integrate meaning‐making, behavioral activation, and emotion regulation strategies aligned with cultural values may enhance treatment outcomes for Puerto Rican individuals.

This study had several limitations. First, examining the PCL‐5 only in English limits generalizability to Spanish‐speaking populations. As many Puerto Ricans are bilingual and learn English in school, future research should examine how language and cultural orientation influence symptom presentation. Second, we did not collect detailed demographic data on language use, trauma history, and cultural factors, which are known to affect PTSD symptom expression (Perilla et al., 2002; Trepasso‐Grullon, 2012). Future work should assess PTSD factor structure across English and Spanish while evaluating cultural and linguistic influences. Third, though this study provides evidence of measurement invariance and structural validity across cultural groups, it does not address clinical utility. Future research should move beyond CFA to examine whether the *DSM‐5* or alternative models (e.g., the *International Classification of Diseases and Related Health Problems* [11th ed.]) differ in predicting treatment response, functional impairment, or diagnostic classification accuracy across cultural groups (Rasmussen et al. 2019; Shevlin et al. 2017). Finally, future qualitative studies could explore how Puerto Ricans conceptualize PTSD and whether culturally specific expressions of distress are underrepresented in Western diagnostic frameworks.

Overall, this study provides strong evidence that the PCL‐5 exhibits comparable factor structure across English‐speaking Puerto Rican and NLW individuals. Puerto Rican communities have faced repeated exposure to natural disasters often accompanied by infrastructure collapse, delayed emergency response, and long‐term financial strain (Canino et al., 2019; Kishore, 2018), which likely exacerbate chronic distress and trauma‐related symptoms. As extreme weather events increase in frequency and severity, Puerto Ricans are likely to remain among the most affected populations (Bakkensen & Ma, 2020). These structural inequities, including environmental injustice, colonial legacies, and limited health care access, shape the way PTSD symptoms are experienced, expressed, and measured over time. To enhance disaster response and trauma care, there is a pressing need for scalable, culturally tailored interventions—including digital tools—to improve outreach, assessment, and continuity of care (Ruggiero et al., 2015).

## AUTHOR CONTRIBUTIONS

**Johanna E. Hidalgo**: Conceptualization; Investigation; Writing – original draft; Methodology; Writing – review and editing; Formal analysis. **Keith B. Burt**: Methodology; Writing – review and editing; Formal analysis. **Tatiana M. Davidson**: Resources; Data curation; Writing – review and editing. **Kenneth J. Ruggiero**: Resources; Data curation; Project administration; Writing – review and editing; Methodology. **Arthur R. Andrews**: Writing – review and editing; Formal analysis; Methodology. **Ateka A. Contractor**: Writing – review and editing; Methodology; Formal analysis. **Kelly Peck**: Writing – review and editing; Methodology. **Ellen W. McGinnis**: Writing – review and editing; Methodology. **Jennifer Ha**: Writing – review and editing. **Natalie C. Noble**: Writing – review and editing. **Julia N. Kim**: Writing – review and editing. **Vanessa Ramirez**: Writing – review and editing. **Matthew Price**: Supervision; Data curation; Methodology; Writing – review and editing; Investigation; Conceptualization.

## AUTHOR NOTE

This research was supported by the National Institute of Mental Health (NIMH; R01 MH107641, R01 MH119193).

All views and opinions expressed herein are those of the authors and do not necessarily reflect those of the funding agency or respective institutions. The authors have no conflicts of interest to report.

## OPEN PRACTICES STATEMENT

The data are not publicly available due to privacy restrictions. The data that support the findings of this study are available upon request from the corresponding author at johanna.hidalgo@uvm.edu.
